# Interpersonal Communication and Maternal Behavioral Practice: A Case Study of Explanatory Causal Machine Learning in Nepal

**DOI:** 10.1016/j.tjnut.2026.101657

**Published:** 2026-06-08

**Authors:** Ben Kelcey, Kenda Cunningham, Edward A Frongillo

**Affiliations:** 1 College of Education, Criminal Justicem Human Services and Information Technology, University of Cincinnati, Cincinnati, OH, United States; 2 Nutrition, Diets, and Health Unit, Helen Keller International, New York, NY, United States; 3 School of Public Health, University of South Carolina, Columbia, SC, United States

**Keywords:** mediation, structural equation modeling, machine learning, Nepal, interpersonal communication, nutrition, program impact paths

## Abstract

**Background:**

There is increasing interest not only in whether a program produced impacts but also the mechanisms through which the impacts were produced. Studies of nutrition interventions have frequently highlighted the role of nutrition education interventions, but few studies have investigated the paths through which those interventions achieve impact.

**Objectives:**

We assessed the total effect of maternal exposure to interpersonal communication with a frontline worker on behavioral practice and the degree to which that effect operated through 2 key pathways: improved awareness and knowledge. We also conducted a comparative case study examining the differences between machine learning (ML) and structural equation model (SEM) approaches.

**Methods:**

We used data from the endline cross-sectional survey of the quasi-experimental Suaahara study of 2040 mothers with a child less than 2 years of age. We estimated the total effect of maternal exposure to interpersonal communication with a frontline worker on her nutrition-related practices and the indirect effects through improvements in awareness and knowledge using ML and conventional SEMs. Models were adjusted for household, child, and maternal factors as well as household location.

**Results:**

Maternal exposure to interpersonal communication with a frontline worker translated into practicing more of the ideal, promoted nutrition-related practices. Both methods suggested a similar total effect—ML: 0.58 (95% CI: 0.37, 0.78) and SEM: 0.60 (95% CI: 0.49, 0.72). ML models, however, attributed a much larger proportion of that total effect to indirect effects operating through awareness and knowledge (ML: 0.24; 95% CI: 0.15, 0.33; SEMs: 0.16; 95% CI: 0.09, 0.21).

**Conclusions:**

Interpersonal communication improved nutrition-related practices, in part due to increasing one’s awareness and knowledge. The disparities in results between ML and SEMs suggested that the production of effects is a more complex, nonlinear or interactive function of awareness, knowledge, treatment and covariates than what is captured by simple linear models.

## Introduction

Maternal and child undernutrition persists as a major global public health burden [1]. Although the world has made progress in reducing stunting, for example, more than 700 million people currently face undernutrition. Projections suggest that, despite progress by some countries related to some targets, achieving the 2030 global nutrition targets is nearly impossible: no country will meet all targets [2]. Conflict, climate change, and, most recently, reductions in global aid will only further increase undernutrition and its consequences. Continued investments, therefore, remain urgent, including those that help unpack what works and does not work, why, how, and for whom.

Maternal education has been shown to be among the most accessible levers for reducing undernutrition [3]. Behavior change brought about through education represents a foundational and necessary channel for improving the nutritional wellbeing of mothers and children [4]. Social and behavior change communication (SBCC) interventions leverage communication, behavior theory, and marketing to promote positive changes in behavior; these interventions have demonstrated promise across a wide range of environments and outcomes [[5], [6], [7], [8], [9], [10], [11], [12]]. One key component of SBCC is interpersonal communication (IPC), direct and personalized interactions between individuals or within small groups, with the dual intention of addressing knowledge gaps and other barriers to adoption of ideal practices, as well as shifting social norms by promoting optimal behaviors. IPC has proven effective across a broad array of domains, populations, contexts, and outcomes. For example, evidence suggests that IPC is effective in improving nutrition and health-related knowledge and practices such as meal preparation, healthy child feeding practices, dietary diversity, and breastfeeding complementary feeding [[13], [14], [15], [16], [17], [18], [19], [20], [21], [22], [23], [24], [25], [26], [27]]. Despite the promise of IPC as a core lever for change, there is a lack of depth and rigor in extant research detailing the specific mechanisms and paths through which IPC interventions produce intended nutrition-related outcomes [23,27,28].

Generating empirical evidence that supports or rebuts theories of behavioral change for nutrition interventions is critical. By examining the processes of how exposure to an intervention changes intermediate variables (e.g., mediators) and in turn influences behaviors, mediation analyses seek to unpack and test components of specific theories underlying an intervention. This process is a critical step in mapping effective interventions and building foundational theory because it provides a comprehensive understanding of the interworking components guiding an intervention while potentially identifying specific breakdowns [29]. For instance, prior research has emphasized the importance of using such intermediate processes to distinguish between instances of implementation failure and theory failure [30]. Implementation failure occurs when an intervention fails to produce changes in a key intermediate mechanism that underlies a theory of change (e.g., when an intervention fails to induce significant changes in knowledge). Theory failure occurs when an intervention changes that intermediate mechanism but those changes do not modify practice (e.g., improved knowledge does not translate into improved practice).

Although theory has regularly suggested that IPC interventions change behaviors by improving nutritional knowledge, research has provided insufficient empirical evidence supporting the mediating or translational role of knowledge [14]. For example, prior studies have largely examined the average treatment effect of IPC interventions focused purely on practice or knowledge without probing how changes in knowledge induced by the intervention translate into changed practice [21]. These knowledge gaps limit our understanding of how IPC interventions drive or fail to drive their intended behavioral changes in nutritional practices and our capacity to improve and adapt the design and implementation of programs to achieve greater results and more efficiently allocate limited social resources.

A growing body of research has also highlighted methodological limitations of conventional methods when examining such complex effects in real-world settings. For example, conventional methods such as structural equation models (SEMs) often take up assumptions (e.g., linearity) that are not well-suited for the complex synergies among mediators and other variables that occur during behavioral change. Recent methodological research has developed more flexible methods that are better suited to such complex settings [31,32]. For example, Bodnar et al. [32] used machine learning (ML) methods to investigate dietary synergy and uncover nonlinear, interactive paths that conventional regression missed. Despite the development of more flexible methods, applications of these methods remain uneven across domains [33,34]. In this study, we examined the average total and indirect effects of an IPC intervention on maternal and child nutrition practices. We examined the degree to which IPC intervention effects operated through improvements in two core mediators that underlie theories of behavior change: awareness and knowledge, using a modern causal ML approach. Furthermore, we compared these direct and indirect effects with those of a conventional linear SEM. Finally, we assessed the degree to which the findings from this observational study are robust to unmodeled or unobserved confounding.

## Methods

### Context

South Asia is heavily burdened by malnutrition, in all its forms. In Nepal, for example, as of the most recent Demographic and Health Survey, 1 in 10 females are of short stature, 1 in 10 underweight and >1 in 3 overweight or obese; among children aged <5 y, 1 in 4 are stunted, nearly 1 in 10 wasted, and ∼1 in 5 underweight [34]. Malnutrition is in part due to poor quality diets with heavy reliance on staple foods. Not even half of Nepalese children 6 to 23 mo consume a minimally acceptable diet, and the prevalence of females of reproductive age consuming a minimally diverse diet was only slightly higher (56%) among females of reproductive age consuming a minimally diverse diet [34]. There is also room for improvement in other behaviors important for ensuring one’s health and nutrition, such as handwashing with soap and water, participating in antenatal and postnatal care, and taking sufficient supplementation during pregnancy and the postpartum period.

**FIGURE 1:**
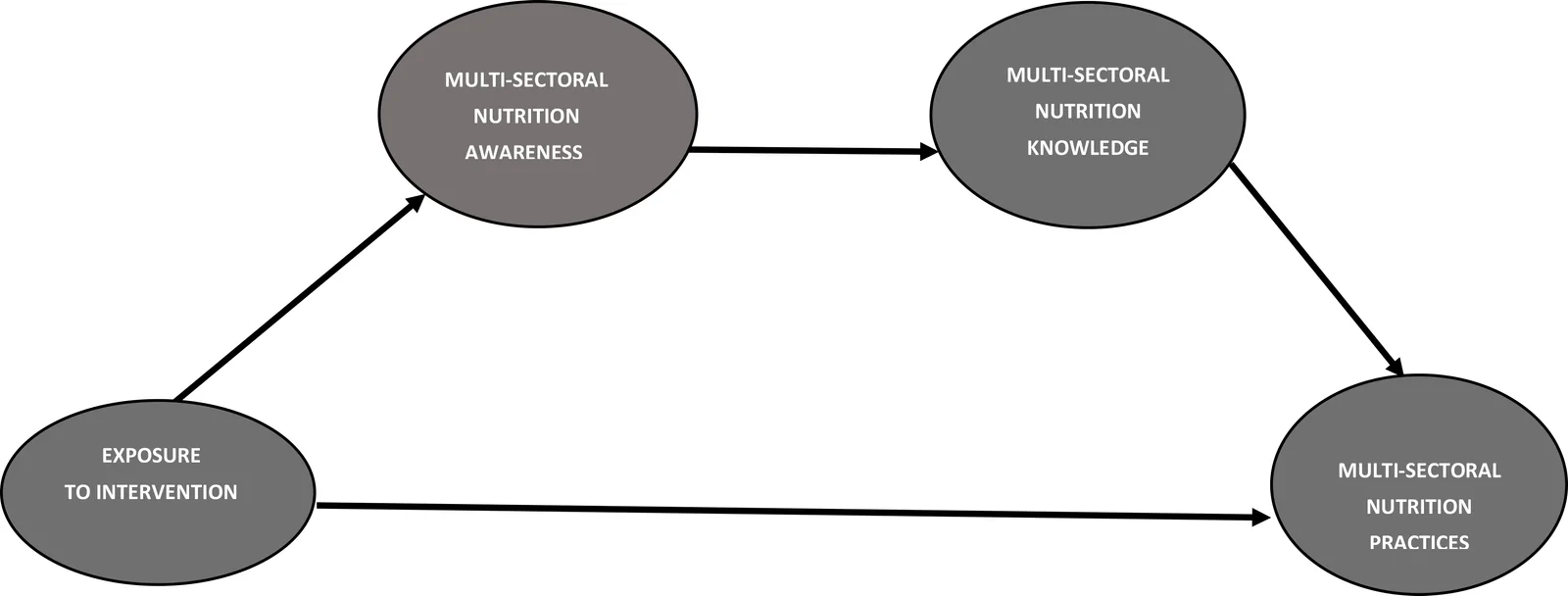
Granular representation of Suaahara II program impact path for social and behavior change interventions.

An at-scale program funded by the United States Agency for International Development, Suaahara, was implemented from 2011 to 2023. Interventions spanning maternal and child health and nutrition, agriculture and food systems, nutrition governance, and water, sanitation and hygiene were designed and implemented by >40 international and local nongovernmental organizations to reduce undernutrition, primarily among mothers and children in the 1000-day period between conception and a child’s second birthday. SBCC interventions consisted of 4 primary delivery channels: *1*) personalized IPC with program frontline workers during home visits and/or phone calls; *2*) community events and demonstrations (e.g., handwashing and cooking) including those facilitated at health mothers’ group meetings led by female community health volunteers; *3*) mass media, including a national edutainment series (Bhanchhin Aama) broadcast via radio and social media; and *4*) short message service messages sent to mobile phones of parents in the 1000-d period. Frontline workers also raised awareness about the other SBCC interventions (community events, mass media, and short message service) and government platforms and encouraged families to engage with them. Suaahara’s program impact path specified how these interventions should translate into outputs, outcomes, and impact (Figure 1). Detailed information on these interventions is published elsewhere [35].

### Design

Our study drew on a quasi-experimental impact evaluation of Suaahara (clinicaltrials.gov identifier NCT05448287) with multistage cluster sampling to randomly sample households across 8 districts (4 intervention and 4 control) to collect baseline (2012) and endline (2022) data in the same communities during the summer monsoon season [35]. Mothers of young children were the primary respondents and male household heads and grandmothers, when available in the household, were secondary survey respondents. These analyses use the full endline dataset with households in both intervention and comparison areas but were limited to those households with a child aged less than 2 years of age (*n* = 2040).

### Data collection

Structured questionnaires were used to collect data after training enumerators and pilot testing each tool. Exposure to IPC (independent variable) was measured by asking each mother whether she had ever been visited at home by or received a phone call from a Suaahara frontline worker. Behaviors of interest (dependent variable) were measured by asking each mother an open-ended questions about her behavior on the following 11 nutrition-related practices, with a binary variable created for each (0, no/1, yes) to indicate whether the correct answer was provided: *1*) participating in ≥ 4 antenatal care visits during pregnancy; *2*) eating more food than usual during pregnancy; *3*) taking ≥ 180 iron and folic acid tablets during pregnancy; *4*) participating in ≥ 3 postnatal care visits during the postpartum period; *5*) taking ≥ 45 iron and folic acid tablets during the postpartum period; *6*) delaying or avoiding getting pregnant by using male or female sterilization, intrauterine device, implants, injectibles, condoms, or birth control pills; *7*) participating in female community health volunteers–led health mothers’ group meetings; *8*) initiating breastfeeding within the first hour of birth; *9*) engaging in appropriate infant and young child feeding of exclusive breastfeeding for those under 6 months of age and introduction of complementary foods between 6 and 8.9 months of age for those 6 to 23 months of age; *10*) treating drinking water by boiling it or using filters, chlorine, or solar; and *11*) always washing hands with soap and water at 6 critical times—after defecation, after cleaning a young child’s bottom, after handling livestock/animals, before cooking/preparing food, before eating, and before feeding children. Similarly, awareness (first mediator) was assessed by asking mothers whether they had ever seen or heard information on those same 11 ideal practices while knowledge (second mediator) was assessed by asking mothers open-ended questions regarding what should be done for each of those 11 ideal practices. Continuous scales (0–11) were created to measure awareness, knowledge, and practices.

### Data analyses

We evaluated the hypothesized program impact path from maternal engagement with Suaahara IPC to maternal and child health and nutrition practices via 2 mediators (awareness and knowledge), adjusted for child age (in completed months); maternal age (in completed years); maternal education (years of schooling); maternal social desirability (sum of 5 questions capturing her overall tendency to answer questions in a manner that is viewed favorably by others); household socioeconomic status (equity score quintiles to assess relative wealth using household asset and housing material data) [36]; caste/ethnicity (excluded caste, Brahmin/Chhetri, and other caste); whether residing in a government-classified disadvantaged community where Suaahara aimed for more intensive interventions; household structure (nuclear or extended); and household location (village). For this evaluation, we defined effects using the potential outcomes framework [37] and used an ensemble of ML algorithms within the targeted learning framework to estimate effects.

To define the effects, we use $M_{i}^{\left(j\right)}\left(t\right)$ as the potential values for mediator *j* (e.g., awareness or knowledge) for individual *i* when exposed to treatment condition $T_{i}=t$. Similarly, with *J* = 2 mediators (awareness and knowledge), we use $Y_{i}\left(t,m_{1},m_{2}\right)$ as the potential outcome (e.g., use of ideal practices) for individual *i* when exposed to treatment condition $T_{i}=t$, and when the mediators are set to the values they would have taken under that treatment assignment (i.e., $M_{i}^{\left(1\right)}=m_{i}^{\left(1\right)}\left(t\right)$ and $M_{i}^{\left(2\right)}=m_{i}^{\left(2\right)}\left(t\right)$). The main or total effect (TE) for individual *i* is defined as follows:

(1) $${TE}_{i}=Y_{i}\left(T_{i}=1,M_{i}^{\left(1\right)}=m_{i}^{\left(1\right)}\left(T_{i}=1\right),M_{i}^{\left(2\right)}=m_{i}^{\left(2\right)}\left(T_{i}=1\right)\right)-Y_{i}\left(T_{i}=0,M_{i}^{\left(1\right)}=m_{i}^{\left(1\right)}\left(T_{i}=0\right),M_{i}^{\left(2\right)}=m_{i}^{\left(2\right)}\left(T_{i}=0\right)\right)$$

The total effect contrasts the potential outcomes associated with switching the treatment conditions (e.g., $T_{i}=1$ or $T_{i}=0$) and the corresponding values of the mediators [e.g., $M_{i}^{\left(j\right)}=m_{i}^{\left(j\right)}\left(T_{i}=1\right)$ or $M_{i}^{\left(j\right)}=m_{i}^{\left(j\right)}\left(T_{i}=0\right)$]. That is, the TE contrasts the potential outcome when the treatment and mediators are set to what they would be under the treatment condition with the potential outcome when the treatment and mediators are set to what they would be under the control condition.

The total natural indirect effect (TIE) contrasts the potential outcomes under exposure when modulating only the mediator values (e.g., $M_{i}^{\left(j\right)}=m_{i}^{\left(j\right)}\left(T_{i}=1\right)$ versus $M_{i}^{\left(j\right)}=m_{i}^{\left(j\right)}\left(T_{i}=0\right)$). The TIE for individual *i* is defined as as follows:

(2) $${TIE}_{i}=Y_{i}\left(T_{i}=1,M_{i}^{\left(1\right)}=m_{i}^{\left(1\right)}\left(T_{i}=1\right),M_{i}^{\left(2\right)}=m_{i}^{\left(2\right)}\left(T_{i}=1\right)\right)-Y_{i}\left(T_{i}=1,M_{i}^{\left(1\right)}=m_{i}^{\left(1\right)}\left(T_{i}=0\right),M_{i}^{\left(2\right)}=m_{i}^{\left(2\right)}\left(T_{i}=0\right)\right)$$

The TIE compares the potential outcomes under exposure to the treatment but with the mediator values set to what they would be under the treatment condition ($T_{i}=1$) compared with the control condition ($T_{i}=0$). In terms of our study, the TIE captures the causal effect of exposure to IPC with a frontline worker on behavioral practices that accrues due to changes in awareness and knowledge.

Similarly, the pure direct effect (PDE) compares the potential outcomes when treatment exposure is modulated between treatment and control conditions while fixing the values of the mediators to what they would have taken under the control condition. The PDE for individual *i* is defined as follows:

(3) $$P{DE}_{i}=Y_{i}\left(T_{i}=1,M_{i}^{\left(1\right)}=m_{i}^{\left(1\right)}\left(0\right),M_{i}^{\left(2\right)}=m_{i}^{\left(2\right)}\left(0\right)\right)-Y_{i}\left(T_{i}=0,M_{i}^{\left(1\right)}=m_{i}^{\left(1\right)}\left(0\right),M_{i}^{\left(2\right)}=m_{i}^{\left(2\right)}\left(0\right)\right)$$

Under these definitions, the TE can be decomposed into the direct and indirect effects as ${TE}_{i}=P{DE}_{i}+{TIE}_{i}$. The average effect for each estimand is then found by contrasting the expectations of the aforementioned potential outcomes.

Within this framework, the effects are identified under several related assumptions. First, identification of the indirect effects requires the observed responses to be consistent with the potential responses under the observed exposure condition. Second, identification requires the stable unit treatment value assumption, which precludes, for example, the treatment assignment of an individual from influencing the potential responses of other individuals [38,39]. Third, identification requires sequential ignorability. This assumption requires the conditional independence of the potential mediator and outcome responses given observed covariates (i.e., no unmeasured confounding), as well as no posttreatment variables that are influenced by treatment exposure that also act as confounders of the outcome–mediator relationship. Identification of the effects also requires positivity or common support between the treatment and control conditions (e.g., covariates do not fully determine treatment condition or mediator values).

### Structural equation modeling

Conventionally, SEMs have been the predominate method to estimating total, direct, and indirect effects [40,41]. SEMs leverage several regression equations to estimate the effects--an equation for each endogenous variable (mediators and outcome) as follows:

(4) $$\;\;\;M_{i}^{\left(1\right)}=\beta_{0}^{M^{\left(1\right)}}+a^{M^{\left(1\right)}}T_{i}+\pi_{k}^{M^{\left(1\right)}}X_{ik}+\varepsilon_{i}^{M^{\left(1\right)}}\;\;M_{i}^{\left(2\right)}=\beta_{0}^{M^{\left(2\right)}}+a^{M^{\left(2\right)}}T_{i}+\pi_{k}^{M^{\left(2\right)}}X_{ik}+\varepsilon_{i}^{M^{\left(2\right)}}\;\varepsilon_{i}^{M}∼MVN\left(0,\Sigma_{M|}\right)\;Y_{i}=\beta_{0}^{Y^{\prime }}+b^{M^{\left(1\right)}}M_{i}^{\left(1\right)}+b^{M^{\left(2\right)}}M_{i}^{\left(2\right)}+c^{\prime }T_{i}+\pi_{k}^{Y}X_{ik}+\varepsilon_{i}^{Y}\;{\;\varepsilon }_{i}^{Y^{\prime }}∼N\left(0,\sigma_{Y|}^{2}\right)$$

Here, we use $M_{i}^{\left(1\right)}$ and $M_{i}^{\left(2\right)}$ as the mediators for individual *i*, $X_{ik}$ as *k* covariates, $Y_{i}$ as the outcome, $\beta_{0}^{Y}$, $\beta_{0}^{M^{\left(1\right)}}$, $\beta_{0}^{M^{\left(2\right)}}\text{,}$and $\beta_{0}^{Y^{\prime }}$ as the intercepts, $\pi_{k}^{.}$ as the respective covariate coefficients, $\varepsilon_{i}^{.}$ as the respective error terms, $a^{M^{\left(1\right)}}$ and $a^{M^{\left(2\right)}}$as coefficients capturing the change in awareness and knowledge induced by program exposure, $b^{M^{\left(1\right)}}$ and $b^{M^{\left(2\right)}}$ as coefficients summarizing how changes in awareness and knowledge are associated with improvements in practices, and *c’* as the direct effect. In turn, we use ${ME}^{SEM}=a^{M^{\left(1\right)}}b^{M^{\left(1\right)}}+a^{M^{\left(2\right)}}b^{M^{\left(2\right)}}$ as an estimate of the mediation or indirect effect with the sum as of the direct and indirect effects as an estimate of the TE (${TE}^{SEM}$).

SEMs provide a simple method for estimating indirect effects. A key concern, however, with defining and estimating mediation effects with parametric models is that they take up unnecessary and often unreasonable assumptions (e.g., linearity, additivity, correct model specification). In the context of mediation, prior literature has widely documented that SEMs return biased estimates if the model does not correctly specify, for example, the parametric family, the full set of nonlinear relationships (e.g., quadratic covariate terms), and the correct set of interactions (e.g., all *n*-way interactions among treatment, covariates, mediators, and their nonlinear counterpart terms) [40,41]. In practice, these assumptions are often difficult to buttress and verify because real-world data may exhibit complex interactions, nonlinearities, and distributions, which do not conform neatly to theoretical distributions, parametric forms, or substantive theories guiding investigations. For instance, theories of action underlying an intervention rarely consider and specify comprehensive sets of interactions (e.g., 3-way interactions) and nonlinearities. Rather, most theories of action offer only granular representations that simply specify directional relationships (Figure 1) rather than the precise functional forms of those relationships. Yet, in many instances, it is plausible that there are unexpected, nonlinear or interactive relationships among the intervention, covariates, and mediators (e.g., mediator–mediator interactions) that exert synergistic, accelerating, or diminishing effects on the outcome [31,42,43].

Prior literature has developed methods that attempt to relax many of these assumptions. For example, SEM literature has developed methods for latent interactions and higher order terms [44]. That same literature has also demonstrated, however, the resulting methods often fall short because they still require correct *a priori* specification of the functional form (e.g., all *n*-way interactions and nonlinearities). Moreover, even when models are correctly specified, the use of model-based (as opposed to model-free potential outcome) definitions of effects (e.g., mediation) can still misrepresent or distort causal effects.

In contrast, the potential outcomes framework supplies model-free definitions of effects that facilitate the use of a wider variety of methods that are often more flexible and rigorous than SEM. Prior literature has demonstrated that many of the assumptions needed for SEM to be valid (e.g., linearity, correct model specification) are unnecessary to identify the mediation effect. Pairing the potential outcomes framework with estimation methods that relax, reduce, or eliminate model assumptions (e.g., non- or semiparametric data-adaptive approaches) can provide more robust estimates and have proven particularly useful when structural models (e.g., propensity score, mediation, or outcome models) are underspecified in theory or practice [31,42]. In some cases, the conventional SEM method and its assumptions can be considered a special application of the potential outcomes framework [31]. For example, when the aforementioned assumptions are fulfilled and there are no interactions, the effects are linear, the mediator, and outcome models are correctly specified, and the outcome and mediators are continuous, literature has demonstrated that mediation effects are identified using SEMs [31]. Pairing the potential outcomes framework with estimation methods (e.g., double robust methods and nonparametric or semiparametric data-adaptive methods) that relax, reduce, or eliminate model assumptions, however, can provide more robust estimates; this has proven particularly useful when structural models (e.g., propensity score, mediation, or outcome models) are underspecified in theory or practice [31].

### Targeted learning

To relax reliance on parametric specifications, we estimated the effects within a targeted learning framework that combined data-adaptive nuisance estimation (based on multiple ML algorithms) with targeted minimum loss estimation (TMLE) [31,45]. Rather than rely on a fallible *a priori* specification of functional relationships, the targeted learning framework draws on data-adaptive methods to empirically estimate a broad range of complex relationships (e.g., nonlinearities and interactions) and subsequently adjust for the degree to which they may confound treatment effects using nuisance models. Nuisance models estimate the functional relationships among, for example, the covariates, treatment, mediators and outcome to account for selection or confounding effects. When investigating indirect effects, for instance, nuisance models include the conditional expectation of the outcome given the treatment condition, mediators, and covariates; the conditional expectation of the mediators given the treatment condition and covariates; and/or the conditional probability of treatment exposure given covariates (i.e., propensity score). In turn, these conditional expectations are used to flexibly predict and contrast potential responses under different treatment conditions.

Despite their flexibility and high predictive ability, data-adaptive methods have the potential to introduce target parameter (e.g., indirect effect) bias because they are built to optimize models for prediction rather than minimizing bias in the target parameter estimate [45]. To address this bias, the targeted learning framework leverages information across models (e.g., outcome model, mediator model, and propensity score model) to iteratively adjust estimates. By combining information across models, TMLE confers a doubly (TE) or multiply (mediation effect) robust property such that it provides consistent estimates of the causal parameter even when one of the nuisance models (e.g., outcome, mediation, or propensity score models) imperfectly captures relationships [45]. This property can be particularly valuable when probing mediation because it ensures estimates remain reliable even if data-adaptive methods do not sufficiently capture all relationships.

We implemented targeted learning using the super learner in the *tmle* package in R software (R Foundation for Statistical Computing) [45]. The super learner is an ensemble ML method designed to enhance predictive accuracy by combining multiple individual ML algorithms or learners. Rather than relying on a single ML algorithm, the super learner leverages the strengths of a diverse set of models that each excel in fitting different data patterns to create more robust and reliable nuisance models. Super learners estimate the performance of each model in the data (e.g., using mean-squared error) and then create optimal weighted averages of those models based on their performances. Cross-validation is used to identify the optimal weight to assign to each algorithm and to mitigate the potential for overfitting. The result is that the weighted combination of learning algorithms performs at least as well as any single learner [45]. This method is particularly valuable in complex models such as mediation where constructing accurate nuisance models and counterfactual outcomes are crucial for valid causal inference. We built the super learner for each of the nuisance models using all the aforementioned covariates and fixed effects for household location (community), ten-fold cross-validation, and the following ML algorithms: generalized additive models, multivariate adaptive regression splines, neural networks, random forests, extreme gradient boosting, elastic-net regularized generalized linear models (lasso and ridge regression), regression trees, and generalized linear models.

### Sensitivity analysis

A key consideration in observational studies is the degree to which the findings are robust to unmodeled or unobserved confounding. In this study, we used several methods to reduce the potential sensitivity of our findings. We used data-adaptive methods with a diverse library of ML algorithms to relax model specification assumptions and protect against, for example, omitted interactions and nonlinear relationships. We also used household location (community) effects to protect against unobserved community-level variables and account for the influence of community composition on the treatment propensity, mediators, and outcome [46]. Similarly, we drew on doubly/multiple robust TMLE estimators to further protect against misspecified models and the influence of unobserved variables.

Nevertheless, the validity of our findings is still subject to the plausibility of the sequential ignorability assumption and the potential for unobserved variable bias introduced through confounding. That is, although we assessed and included many core variables that are associated with maternal nutrition awareness, knowledge, and practice, the scope of our covariate controls is still fallible. To probe the sensitivity of our results, we assessed the degree to which our results would be altered by additional adjustment for unobserved covariates. We benchmarked the range of bias introduced by potential unobserved variables by empirically evaluating the bias introduced by omitting each of the observed covariates. We outlined the results by reporting the adjusted indirect effects and CIs when omitting each observed covariate. We also report the implied indirect effect (and CI) that outlines the resulting altered effect had we controlled for an unobserved covariate that had comparable relationships. The original indirect effect was considered robust to violations of the sequential ignorability if the confidence interval for the implied indirect effect excluded a null effect.

### Ethics

The impact evaluation endline survey received ethical approval (Reg. no. 236/2021) from the Nepal Health Research Council. All participants provided written informed consent to participate in the study and were reminded periodically throughout the survey that they could discontinuation participation for any reason, without consequence.

## Results

### Sample characteristics

In the study sample, children were 12 months of age, on average, and their mothers were, on average, 26 years old. Approximately one-fifth of the sampled mothers had not completed at least primary school. Slightly less than half of these households were from the upper Brahmni/Chhetri caste groups. About one-third of the mothers had been exposed to Suaahara IPC (*n* = 677). For the eleven key nutrition-related behaviors assessed in this study, on average, mothers were aware of about nine of them, reported accurate knowledge on almost seven of them, and practiced almost five of them (Table 1).

**TABLE 1:** Maternal sociodemographic characteristics, exposure to Suaahara II interpersonal communication, and nutrition-related awareness, knowledge, and practices

Characteristic	Value, *N* = 2040, mean/proportion (SD)
Household characteristics	
Household resides in disadvantaged community	0.22
Extended/nuclear household	0.57
Caste/ethnicity	
Socially excluded	0.47
Brahmin/Chhetri	0.40
Other	0.12
Socioeconomic quintiles	
Poorest (household socieconomic quintile 1)	0.16
Second poorest (household socieconomic quintile 2)	0.20
Middle (household socieconomic quintile 3)	0.19
Second wealthiest (household socieconomic quintile 4)	0.31
Wealthiest (Household socieconomic quintile 5)	0.14
Child and maternal characteristics	
Child age (mo)	12.01 (6.96)
Maternal age (y)	25.62 (5.22)
Maternal social desirability	31.45 (3.86)
Maternal education	
No school	0.10
Some primary school	0.09
Completed primary (grade 5)	0.06
Some secondary	0.34
Completed secondary (grade 10)	0.20
Completed grade 12	0.18
Higher education	0.03
Outcome, exposure, and mediator variables	
Exposure to interpersonal communication	0.33
Awareness of key messages/information	9.13 (1.76)
Knowledge of ideal practices	6.93 (1.86)
Ideal practices	4.94 (1.66)

Descriptive statistics by maternal exposure status to Suaahara II IPC and their unconditional mean differences are outlined (Table 2). In terms of pretreatment covariate balance, the composition of individuals exposed to IPC with a frontline worker tended to demonstrate more privilege than those in the control condition. Those exposed to IPC with a frontline worker tended to be from higher socioeconomic quintiles, more educated, from the Brahmin/Chhetri caste, and residing in nondisadvantaged communities. In terms of mediators and outcomes, exposure to IPC with a frontline worker was associated with unconditional gains of 1.05 in awareness, 0.79 in knowledge, and 0.91 in practices.

**TABLE 2:** Unconditional means (SDs) in covariates, mediators, and outcome between mothers who were exposed vs. unexposed to Suaahara II interpersonal communication interventions

Variable name	Treated (*n* = 677)	Control (*n* = 1363)	Mean difference (SE)	*P*∗ value
Household socioeconomic status	3.22 (1.26)	3.00 (1.33)	0.22 (0.06)	<0.01
Socially excluded caste	0.39 (0.49)	0.51 (0.50)	−0.13 (0.02)	<0.01
Brahmin/Chhetri caste	0.18 (0.38)	0.10 (0.30)	0.08 (0.02)	<0.01
Child age (mo)	12.30 (6.83)	11.87 (7.02)	0.43 (0.33)	0.19
Maternal age	25.69 (5.00)	25.58 (5.34)	0.11 (0.25)	0.66
Maternal education	4.31 (1.52)	4.00 (1.60)	0.32 (0.07)	<0.01
Joint/nuclear household	0.65 (0.48)	0.53 (0.50)	0.12 (0.02)	<0.01
Maternal social desirability	31.65 (3.85)	31.36 (3.87)	0.29 (0.18)	0.11
Household resides in disadvantaged community	0.50 (0.50)	0.09 (0.28)	0.41 (0.02)	<0.01
Awareness of key messages/information	9.83 (1.27)	8.78 (1.87)	1.05 (0.08)	<0.01
Knowledge of ideal practices	7.45 (1.62)	6.67 (1.92)	0.79 (0.09)	<0.01
Ideal practices	5.55 (1.61)	4.63 (1.60)	0.91 (0.08)	<0.01

∗ *P* < 0.05.

The total, direct, and indirect effects are summarized by estimator, after adjustment for household socioeconomic status, caste/ethnicity, child age, maternal age, maternal education, maternal social desirability, the number of people in the household, whether or not the household resides in a government-identified disadvantaged community, household structure (nuclear or extended), and household location (community) in Table 3. Maternal engagement with frontline workers for IPC and summative practice was *TE* = 0.60 (95% CI: 0.49, 0.72) under TMLE and ${TE}^{SEM}$ = 0.58 (95% CI: 0.37, 0.78). That is, maternal exposure to IPC with frontline workers increased adoption of ideal practices by just above a half of a behavioral practice. In terms of a standardized effect, maternal exposure to IPC with frontline worker increased use of ideal practices by 0.36 (TMLE) and 0.35 (SEM) standard deviations.

**TABLE 3:** Effects by estimator and approach

Effect	TMLE		LM/SEM	
	Estimate	CI	Effect	CI
Direct	0.36	[0.18, 0.54]	0.42	[0.23, 0.62]
Indirect	0.24	[0.15, 0.33]	0.16	[0.09, 0.21]
Total	0.60	[0.49, 0.72]	0.58	[0.37, 0.78]

Abbreviation: LM, linear model; SEM, structural equation modeling; TMLE, targeted minimum loss estimation.

Although TMLE and SEM largely agreed on the TE and its uncertainty, they substantively differed on how that effect was achieved. Decomposition of that TE with SEM suggested around 27% (${ME}^{SEM}=$0.16; 95% CI: 0.09, 0.21) of the TE operated through differences in awareness and knowledge with the direct effect accounting for the remaining 73% (*c’* = 0.42; 95% CI: 0.23, 0.62) of the TE. By contrast, TMLE indicated that the indirect effect of greater awareness or knowledge induced by maternal exposure to IPC with a frontline worker explained 40% of the TE (TIE = 0.24; 95% CI: 0.15, 0.33) with the direct effect accounting for 60% (PDE = 0.36; 95% CI: 0.18, 0.54) of the TE. TMLE, thus, suggested a different decomposition of the TE than that suggested by SEM. Under TMLE, the indirect effect was about 50% larger (TIE = 0.24) than that traced by SEM (${ME}^{SEM}=$0.16).

The contrast of cross-validated mean-squared errors between the approaches suggested that the flexible model form adopted in TMLE explained more variance in awareness, knowledge, and practice than linear SEM (Table 4). For example, elastic-net regression, generalized additive models, and random forests tended to outperform linear SEMs in terms of model-data fit. When the ML algorithms were combined into the super learner, the results demonstrated that the super learner provided mild but uniform improvements over linear SEMs (and other estimators). For example, the super learner improved on SEMs by reducing the average mean-squared error by about 2% for awareness, 2% for knowledge, and 4% for practice (Table 4). In terms of variance explained, the super learner explained around 4%, 5%, and 8% more variance in awareness, knowledge, and practice than SEMs. Collectively, the cross-validation results suggested TMLE better captured relationships among covariates, mediators, and the outcome because it was able to track nonlinear and interactive relationships.

**TABLE 4:** Cross-validated mean-squared error (MSE) by learner for mediators and outcome

Machine learning algorithm	Awareness		Knowledge		Practice	
	Average	SE	Average	SE	Average	SE
Super learner	2.08	0.08	2.41	0.07	1.85	0.06
Regression trees	2.60	0.10	3.11	0.10	2.10	0.07
Random forests	2.17	0.08	2.54	0.08	1.87	0.06
Generalized additive models	2.12	0.08	2.45	0.08	1.91	0.06
Extreme gradient boosting	2.32	0.09	2.64	0.08	2.03	0.06
Lasso/elastic-net	2.10	0.08	2.44	0.07	1.89	0.06
Multivariate adaptive regression splines	2.58	0.15	3.22	0.25	2.15	0.07
Neural networks	2.97	0.11	3.40	0.11	2.75	0.08
Generalized linear models/structural equation models	2.12	0.08	2.46	0.08	1.92	0.06

The results of the the sensitivity analysis in terms of potential confounding bias suggested that our inference regarding the indirect effect was largely robust to the omission of an unobserved variable if that variable was comparable with a covariate in our original collection (Table 5). Omitting maternal social desirabilitychanged the indirect estimate from 0.24 to 0.29 and suggested that omission of a comparable unobserved covariate might reduce the original indirect estimate from 0.24 to 0.19. The corresponding CI still excluded a null effect (95% CI: 0.10, 0.27) and still supported the impact of IPC on practice operates through awareness and knowledge. Omission of the household location (community) effects demonstrated the largest potential: had we omitted such indicators, the indirect would have increased from 0.24 to 0.39 (95% CI: 0.27, 0.51). Omission of a comparable but unobserved covariate or set of covariates might reduce the original indirect estimate from 0.24 to 0.09. The corresponding confidence interval still (narrowly) excluded a null effect (95% CI: 0.00, 0.16) and provided support for mediation. The sustained statistical confidence despite the point estimate change observed when omitting household location (community) effects not only outlines the robustness of the results but also highlights the importance of adjusting for differences among communities in terms of their compositions and processes.

**TABLE 5:** Sensitivity analysis: indirect effect and CIs when covariate is omitted (adjusted indirect effect) and the implied indirect effect and CIs assuming a comparable covariate was omitted from the original analysis (implied indirect effect)

Covariate	Adjusted indirect effect	Adjusted CI	Implied indirect effect	Implied CI
Household socioeconomic quintile	0.21	[0.13, 0.29]	0.27	[0.18, 0.36]
Caste	0.21	[0.12, 0.29]	0.27	[0.18, 0.36]
Child age (mo)	0.18	[0.09, 0.27]	0.30	[0.21, 0.39]
Maternal age	0.19	[0.10, 0.27]	0.29	[0.20, 0.38]
Maternal education	0.25	[0.17, 0.33]	0.23	[0.14, 0.32]
Joint/nuclear household	0.25	[0.17, 0.33]	0.23	[0.14, 0.32]
Maternal social desirability	0.24	[0.15, 0.33]	0.24	[0.15, 0.33]
Household resides in disadvantaged community	0.23	[0.15, 0.32]	0.25	[0.16, 0.34]
Community	0.39	[0.27, 0.51]	0.09	[0.00, 0.16]

Note: The adjusted indirect effect describes how the indirect effect changed when the corresponding covariate was omitted, whereas the implied indirect effect describes how the original estimate of the indirect effect would change had we omitted an unobserved covariate that was comparable with the corresponding observed covariate.

Abbreviation: CI, 95% confidence interval.

## Discussion

This study investigated the translation of IPC interventions on nutrition practices directly and through improvements in awareness and knowledge in Nepal. Maternal exposure to Suaahara II frontline workers for IPC had a TE of 0.60 (95% CI: 0.49, 0.72) on ideal, promoted nutrition-related practices. The theory of change embedded in the program was supported using both SEM and ML methods. ML methods, however, attributed a much larger proportion of the TE to the indirect path of awareness and knowledge (0.24; 95% CI: 0.15, 0.33) than did SEMs (0.16; 95% CI: 0.09, 0.21). Sensitivity analyses also suggested the results were reasonably robust to unobserved confounding variables, if they were comparable with the observed set.

Results from these analyses are consistent with other recent studies, suggesting the value of IPC for improving nutrition-related knowledge and/or practices. For example, a recent study in rural Indonesia found maternal exposure to IPC to be associated with improved IYCF knowledge and separately found a positive association between maternal knowledge and Infant and Young Child Feeding (IYCF) practices [32]. Studies of the well-known Alive and Thrive interventions found any exposure to IPC to be associated with 2 times increased odds of exclusive breastfeeding in Vietnam and that exposure to IPC 4 to 6 times in Bangladesh tripled the odds of exclusive breastfeeding, highlighting that the frequency of IPC contacts necessary to achieve IYCF-related behavior change may vary by context [47]. Although our analyses focused on a single intervention (IPC) controlling for other social and behavior change and systems-level interventions, reviews have shown that a combination of different types of interventions (i.e., individual- and group-level interventions) using different platforms/locations (i.e., homes, communities, and health facilities) tend to amplify effects compared with single intervention [48]. Thus, the effect size attributed to IPC interventions by Suaahara frontline workers capture only 1 component of effects and the collective effects owing also to other social and behavior change and systems-level interventions are likely to be much larger.

From an analytic perspective, the disparity between TMLE and SEM indirect effect estimates despite the similarity of their TE estimates raises the possibility that the SEM was mildly misspecified. For example, although SEM may provide a reasonable approximation of the TE relationships (i.e., treatment, covariates, and practice), sublte nonlinearities and/or interactions involving the mediators may have been missed by SEM but tracked by TMLE. The difference between the performance of nuisance models estimated with SEM and TMLE also supported the presence of such mediator relationships. The difference in (cross-validated) average mean-squared error when modeling practice as a function of the covariates and treatment was only 0.03 (2.06 for TMLE and 2.09 for SEM)—that is, TMLE offered only a 1% improvement over SEM for the TE model. By contrast, when modeling practice as a function of the mediators, covariates, and treatment, the differences between TMLE and SEM in terms of the (cross-validated) average mean-squared error increased (1.85 for TMLE and 1.92 for SEM)—that is, TMLE offered a 4% improvement over SEM. Taken together, the results suggested that the data-adaptive methods were mildly better suited to track the complexity of the mediator relationships with covariates, each other (e.g., interactions) and the outcome than the SEM.

The disparities between the ML and SEM results also suggested more practical implications. Most theories underlying interventions offer only coarse sketches of the relationships among covariates, an intervention, an outcome, and the mediating variables. Implicit, however, are the possibilities of complex interactions and synergistic relationships. For instance, prior literature has suggested that complex interactions among, for example, awareness, knowledge, and attitudes may frequently shape behaviors. In the current context, for instance, maternal awareness of the need to treat drinking water and knowledge of specific and appropriate treatments may critically interact to shape water treatment practices. Similarly, awareness that one should exclusively breastfeed to achieve health benefits and detailed knowledge on how often, until when, and how to address breastfeeding challenges to achieve exclusive breastfeeding may cooperate in complex ways. That is, there may be a nonadditive or synergistic effect that arises only when mothers are both aware of the importance of a specific practice and have specific knowledge of how to adopt that practice. Alternatively, there may be interactions between awareness and knowledge that exhibit compensatory effects. For example, having only awareness or knowledge of handwashing with soap may be sufficient for promoting the regular practice of handwashing.

Despite the plausibility of such interactions, the empirical specification and investigation of such interactions through quantitative analysis are almost imperceptible in literature. In part, the absence of such empirical research may owe to the limitations of conventional methods. Conventional methods largely presume no interactions and even when interactions are considered, models typically introduce only one or two interactions because it is often intractable to probe and interpret all potential *n*-way interactions. In contrast, the data-adaptive nature of ML methods can facilitate discovery of such interactions and other complex functional relationships. The results of our comparison between ML and SEM methods further suggested that ML methods may be better suited to capture the production of treatment effects even when the theory of action is not precisely specified in exhaustive detail.

## Author contributions

The authors’ responsibilities were as follows—All authors: designed research, conducted research, analyzed data, wrote the paper, had primary responsibility for final content, and read and approved the final manuscript.

## Data availability

Data described in the manuscript, code book, and analytic code will be made available upon request pending approval by Helen Keller International.

## Declaration of Generative AI and AI-assisted technologies in the writing process

The authors declare that no generative AI or AI-assisted technologies were used in the writing of this manuscript.

## Funding

This work is funded through the Innovative Methods and Metrics for Agriculture and Nutrition Action (IMMANA) program, led by the London School of Hygiene & Tropical Medicine (LSHTM), in partnership with Tufts University and the University of Sheffield. IMMANA is cofunded with United Kingdom Aid from the United Kingdom government and by the Gates Foundation INV-002962/OPP1211308. Under the grant conditions of the Foundation, a Creative Commons Attribution 4.0 Generic License has already been assigned to the author accepted manuscript version that might arise from this submission. Please note works submitted as a preprint have not undergone a peer review process.

## Conflict of interest

The authors report no conflicts of interest.
